# Latex Fruit Syndrome as a Case of a Lower GI Bleed

**DOI:** 10.7759/cureus.65002

**Published:** 2024-07-20

**Authors:** Samrawit W Zinabu, Ahmad Mohammed, Girma M Ayele, Rohan Kuruvilla, Miguel Ramall, Emmanuel Kerolle, Miriam B Michael

**Affiliations:** 1 Internal Medicine, Howard University Hospital, Washington DC, USA; 2 Nephrology, Howard University Hospital, Washington DC, USA

**Keywords:** ige (immunoglobulin e), fruit allergy, oral allergy syndrome, fruit latex syndrome, latex allergy

## Abstract

Latex-fruit syndrome is characterized by hypersensitivity reactions to certain plant-derived foods in individuals allergic to natural rubber latex (NRL), affecting approximately 30-50% of NRL-allergic patients. This condition arises due to the cross-reactivity of IgE antibodies. Over time, this syndrome has been associated with an increased number of plant sources, including avocado, banana, chestnut, kiwi, peach, tomato, potato, and bell pepper. We present a case of an art student who developed latex-fruit syndrome following prolonged exposure to NRL art supplies.

## Introduction

Latex fruit syndrome is a condition where individuals who are sensitive or allergic to natural rubber latex (NRL) also experience allergic reactions to certain fruits and vegetables. These fruits are banana, avocado, chestnut, kiwi, and papaya. People with natural latex allergies who take one of these fruits can develop systemic anaphylaxis, urticaria, angioedema, and oral allergy syndrome [1].

In most cases, NRL allergy precedes fruit allergy. Still, in a few instances, fruit allergy may come first or present simultaneously associated with hypersensitivity, especially to freshly consumed fruits [1]. This association is due to IgE antibodies and usually presents as a cutaneous reaction with swelling, numbness, tingling, and rare anaphylaxis. However, many individuals with latex fruit syndrome present with gastrointestinal symptoms, including nausea and diarrhea [2]. There is no cure for food allergy, and allergic subjects are compelled to extreme avoidance of suspected allergenic foods [3].

## Case presentation

A 21-year-old female with a past medical history of multiple food allergies presented to the emergency room with a one-week history of abdominal pain. This abdominal pain, associated with food intake, manifested as a gnawing discomfort in the epigastric area, rectal pain, and cramping loose stools. She also experienced occasional bright red blood per rectum and, on occasion, clots. The bleeding was spotty and infrequent, occurring during bowel movement. She reported loose stools but not watery diarrhea, typically associated with cramping pain and discomfort.

The patient stated being careful about her allergies, which had become more symptomatic in the past month, leading her to avoid bananas, avocados, and most tropical fruits (pineapples, mangoes, and papayas). However, her roommate recently bought a box of kiwis, and she felt these would be safe since they are from New Zealand. She consumed several kiwis over the past week. She denied any tingling, swelling, or numbness when eating any food in the last week. She also had no history of fever, chills, headaches, photophobia, or chest pain. She was a student at an Art Institute who had recently completed her sculpting module, working with clay and plaster and creating molds from figurines using natural latex. She does not drink, smoke, or use illicit drugs.

On physical examination, she was alert and oriented, answering questions concisely. Physical findings were significant only for mild epigastric tenderness. There was no rebound, guarding, masses, or costovertebral angle (CVA) tenderness. Laboratory investigations revealed that her stool was guaiac-positive, her white blood cell (WBC) count was 5.3 x 10³/µL, and her hematocrit and hemoglobin levels were 33% and 10.9 g/dL, respectively, with all red blood cell indices within the reference range. Blood chemistry values, including sodium at 140 mEq/L and potassium at 4.0 mEq/L, were also within normal limits. The patient was admitted, given intravenous fluids, monitored for further bleeding, cramping, and loose stools, and was discharged the next day with a follow-up in the allergy clinic.

## Discussion

NRL allergies are thought to occur in up to 4.2% of people worldwide. As a result of the protective gear used in the medical field, healthcare workers have a high risk of exposure to latex; it is estimated up to 13% of these individuals are sensitized to latex. This rate has sharply declined in the last 20 years due to reduced natural latex used in manufacturing, medical supplies, and goods. Latex allergies are not always a result of direct contact with latex, as latex syndrome can also occur through consuming certain foods. Fruit Latex Syndrome is a specific phenomenon that correlates NRL hypersensitivities to certain food hypersensitivities and is similar to oral allergy syndrome [4].

The increased use of latex gloves and condoms in 1980 due to the AIDS epidemic worldwide has been proposed to explain the sudden increase in the number of persons affected by latex allergy, starting in the 1980s and extending into the 1990s [5]. Two other factors that could have played a role in increasing latex allergy were an increase in the allergen content of gloves due to the selective growth of higher latex-yielding trees and the use of chemical agents on trees to enhance their yield [6]. Some yield enhancers used on rubber trees are ethylene compounds, and similar ethylene compounds are used to ripen fruit artificially [3,6]. Food allergies are regulated by cross-link reactions between specific food proteins (the allergens) and IgE. However, at the moment, there is still an incomplete understanding of the structural characteristics.

The first documented case of latex-fruit allergy was reported in the early 1990s, involving a patient with a known history of natural latex allergy and a specific food allergy to bananas. This association was established by a radioallergosorbent test (RAST)-inhibition assay [6]. This blood test detects specific IgE antibodies to determine the substance a subject is allergic to and has the advantage of being highly specific with good reproducibility. It was in 1994 that the true relationship was proposed based on the observation from 25 latex allergy patients who developed an increased rate of fruit sensitivity [7]. 

The frequency of fruit and latex allergy occurring simultaneously ranges from 21% in a study done by Kim and Hussain in 1999 to 58% in a study done by Lavaud et al. in 1995. Additionally, one case-control study done by Brehler et al. compared the prevalence of food allergy in 150 patients with a known latex allergy and 30 people without a history of latex allergy and concluded that of the case group, 42.6% had a reaction to different types of fruits like kiwi and bananas. Gastrointestinal and malaise symptoms were the most commonly reported; eight patients developed asthma, and four developed anaphylactic shock [8].

**Figure 1:**
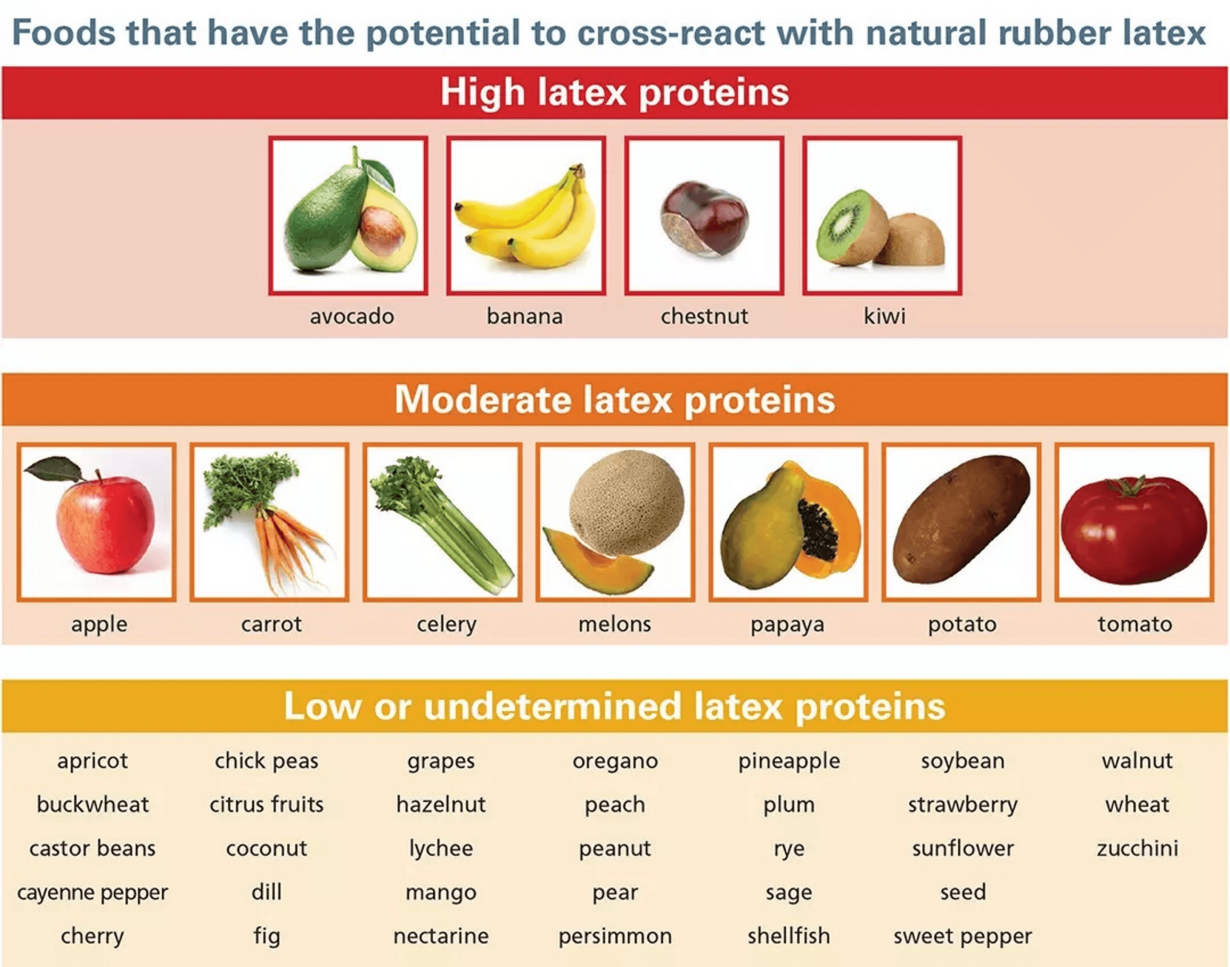
Foods that have the potential to cross-react with natural rubber latex. Credit: Image used with permission from Allergy & Asthma Network [4].

The most commonly implicated fruits in this syndrome (Figure 1) are bananas, kiwis, chestnuts, and avocados [9]. These fruits contain proteins structurally similar to latex, which can trigger cross-reactivity in individuals hypersensitive to latex. In addition to the primary factors, several other fruits have been identified as less common triggers of latex fruit syndrome. These include tomato, pineapple, passion fruit, mango, fig, and papaya (Figure 1) [8].

The pollen-food allergy syndrome is characterized by the association between certain IgE-mediated sensitizations to aeroallergens and various food hypersensitivities, such as birch pollen with Rosaceae fruits, melons with ragweed, and celery with mugwort [7]. The immunopathologic basis for these clinical associations can be attributed to the cross-reactivity among antigens from different sources, which results from molecular similarities among their epitopes [9,10]. However, it is important to note that in vitro cross-reactions may sometimes produce false-positive results of specific IgE tests, which can lead to a confusing diagnosis and should be carefully evaluated [10].

Although latex allergy precedes food hypersensitivity in most patients, the opposite can also be seen. Kenneth Kim from the University of California-Los Angeles (UCLA) found that in 11 of the 29 patients in his study, the food allergy was present before the latex allergy, and 1 patient reported a simultaneous onset [5,9]. There is also evidence that most patients with latex allergy had a history of atopy. This is further supported by Brehler et al. in a study from Germany showing 63% of patients manifesting atopic disease (allergic rhino-conjunctivitis, allergic asthma, and atopic eczema), and 67% had increased total serum IgE levels (>100 kU/1) [9,11].

The allergic reaction induced by Latex Fruit Allergy is likely to stem from cross-reactivity syndrome as well as traditional direct sensitization. This hypothesis is supported by the known cross-reactivity of a group of latex allergens. Hevin is a protein that has been identified as a significant allergen for patients who are allergic to the latex extracted from the rubber tree *Hevea brasiliensis* [11]. Moreover, hevein is the primary allergen chiefly responsible for the cross-reactivity syndrome [8,9] of both plant and insect chitinases. Hev B6.02 is the pro-precursor protein of hevein, renowned for its cross-reactivity with defense-related plant proteins, the Class I chitinases [12]. It has been reported that plant chitinases exhibit a structural similarity with hevein. Additionally, it has been found that an edible insect allergenic chitinase has a structural similarity with an allergenic chitinase from the house dust mite *Dermatophagoides* *farinae* [3].

Chitinases are enzymes that degrade chitin, a substance found in the outer skeleton of insects, fungi, yeasts, algae, crabs, shrimps, lobsters, and other invertebrates' internal structures. Plants use chitinase as a defense system against major constituents of fungal walls and arthropod exoskeletons [13]. Notably, inactivating class I chitinases by heating could explain why plant foods containing these putative allergens consumed after cooking are not associated with the syndrome. 

Another explanation for the growing trend of plant food association with latex allergy can be found in the use of ethylene oxide, a chemical frequently used to speed up ripening. This substance is a known inducer of Class I chitinase expression and could be partly responsible for the growing prevalence of latex-fruit allergy [8].

It is important to consider the physiological role of chitinases as pathogenesis-related proteins. When stressed or attacked by pathogens, they have increased expression [14], and with the introduction of further climate change-related stressors in recent years, there are more stressors impacting organisms. These additional stressors may further increase the production of chitinases. For individuals who are allergic to chitinases, this increased presence in the environment could pose a heightened risk, potentially exacerbating their allergic reactions.

## Conclusions

Food allergies result from an immune reaction between specific food proteins, referred to as allergens, and IgE. Currently, there is no known cure for food allergies, necessitating stringent measures for allergic individuals to avoid allergenic and potentially allergenic foods. Chitinases that cause food allergies are a small group of proteins, but their significance as allergens is notable because they are present in commonly consumed fruits and plant products.
